# Schedule feasibility and workflow for additive manufacturing of titanium plates for ranioplasty in canine skull tumors

**DOI:** 10.1186/s12917-020-02343-1

**Published:** 2020-06-06

**Authors:** J. James, M. L. Oblak, A. R. zur Linden, F. M. K. James, J. Phillips, M. Parkes

**Affiliations:** 1 Department of Clinical Studies, Ontario Veterinary College, University of Guelph, Guelph, Ontario Canada; 2 College of Arts, University of Guelph, Guelph, Ontario Canada; 3 Centre for Advanced Manufacturing and Design Technologies (CAMDT), Sheridan College, Brampton, Ontario Canada; 4 Additive Design in Surgical Solutions Centre (ADEISS), London, Ontario Canada

**Keywords:** Dog, Cancer, Oncology, 3d print, Additive manufacturing, Implant, Cranioplasty

## Abstract

**Background:**

Additive manufacturing has allowed for the creation of a patient-specific custom solution that can resolve many of the limitations previously reported for canine cranioplasty. The purpose of this pilot study was to determine the schedule feasibility and workflow in manufacturing patient-specific titanium implants for canines undergoing cranioplasty immediately following craniectomy.

**Results:**

Computed tomography scans from patients with tumors of the skull were considered and 3 cases were selected. Images were imported into a DICOM image processing software and tumor margins were determined based on agreement between a board-certified veterinary radiologist and veterinary surgical oncologist. Virtual surgical planning was performed and a bone safety margin was selected. A defect was created to simulate the planned intraoperative defect. Stereolithography format files of the skulls were then imported into a plate design software. In collaboration with a medical solution centre, a custom titanium plate was designed with the input of an applications engineer and veterinary surgery oncologist. Plates were printed in titanium and post-processed at the solution centre. Total planning time was approximately 2 h with a manufacturing time of 2 weeks.

**Conclusions:**

Based on the findings of this study, with access to an advanced 3D metal printing medical solution centre that can provide advanced software and printing, patient-specific additive manufactured titanium implants can be planned, created, processed, shipped and sterilized for patient use within a 3-week turnaround.

## Background

Canine cranial tumors are often challenging to treat due to complex regional anatomy and reconstruction. Historically in dogs, closure of the skull defect included a temporalis muscle or fascia flap with or without a polymethylmethacrylate cap [1–3]. More recently, the use of titanium mesh has been described. Titanium mesh for canine cranioplasty is easy to use and has a good cosmetic outcome with limited complications, but surgical time may be prolonged due to the need to contour the implant intraoperatively [1, 4, 5]. The use of additive manufacturing for preoperative printing of patient-specific titanium implants has the potential for significant impact in canine cranial reconstruction.

The emergence of additive manufacturing technology has allowed for the development of patient-specific implants and cutting guides to assist in both the pre and intraoperative phases. Patient-specific additive manufactured implants have been described for many applications in human patients including the correction of dental and maxillofacial deformities [6, 7]. The use of this technology reduces surgery time, speeds healing, and improves clinical outcome [6, 8, 9]. In veterinary medicine, reports of additive manufacturing in surgery are limited to case reports or experimental studies, including the creation of a customized surgical plate for canine tibial plateau leveling osteotomy, correction of a persistent hard palate defect, the production of titanium mesh cages and plates (imbued with repair stimulating substances) to repair canine radial defects, and most recently a feline titanium mandibular prosthesis [9–12]. The timeline associated with each study were either not reported or had a greater than 4-week turnaround from diagnosis until surgery [9–12].

The aim of this feasibility study was to solidify a workflow and schedule for additive manufacturing titanium implants for surgery that can be applied to various procedures involving cranial surgery. To the author’s knowledge, this is the first report in the scientific literature regarding the workflow for metallic additive manufacturing for cranioplasty in veterinary medicine.

## Results

Three patient CT scans were selected for inclusion in this feasibility study.

### Patient 1

**Fig. 1:**
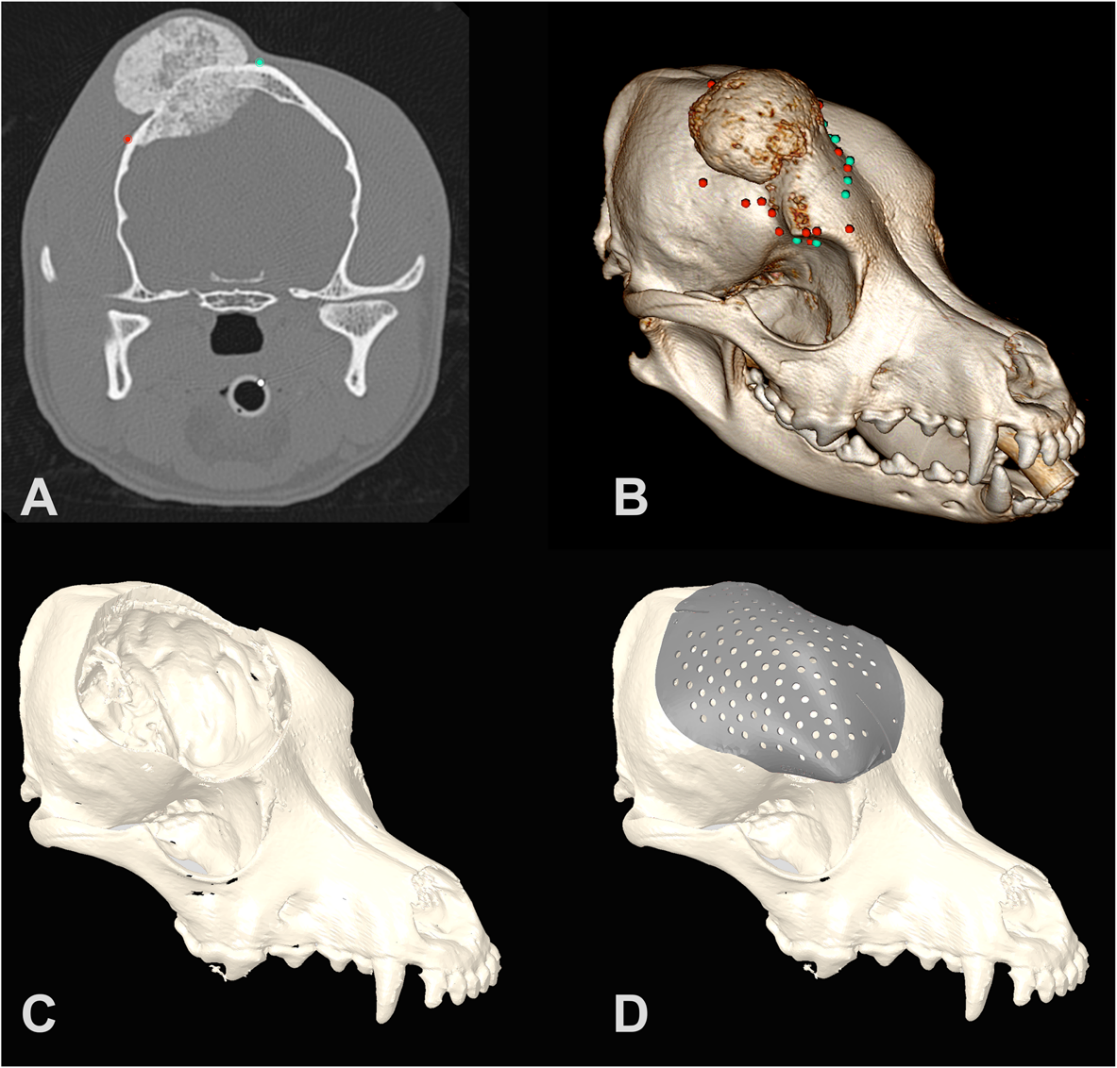
**a.** CT image with Region of Interest (ROI) points for Patient 1 in OsiriX MD imaging software. **b.** Three-dimensional reconstruction with ROI points in OsiriX MD. **c.** Three-dimensional spline in Geomagic Freeform software with tumor removed based on surgical cutting margins. **d.** Titanium plate designed in ADEPT software

Two-year-old, castrated male Bichon Frise with a large bony calvarial mass that was present for 1 year and 5 months prior to imaging. The CT scan revealed a 28 mm H × 33 mm W × 51 mm L expansile bony mass centered on the junction between the right frontal and parietal bones (Fig. 1a). Destruction of the underlying bone was noted. The mass was smoothly marginated with some lobulation and had a coarse, stippled internal architecture. The mass extended into the cranial vault by 16 mm and ventrally compressed the underlying right parietal and frontal lobes with an adjacent hypodensity of the cerebral gray matter consistent with edema and left sided shift of the falx cerebri. The mass also extended rostrally into the right frontal sinus, filling the caudal two-thirds. There was no evidence of abnormal contrast enhancement of the mass or the brain. The histologic diagnosis was an osteoma.

### Patient 2

**Fig. 2:**
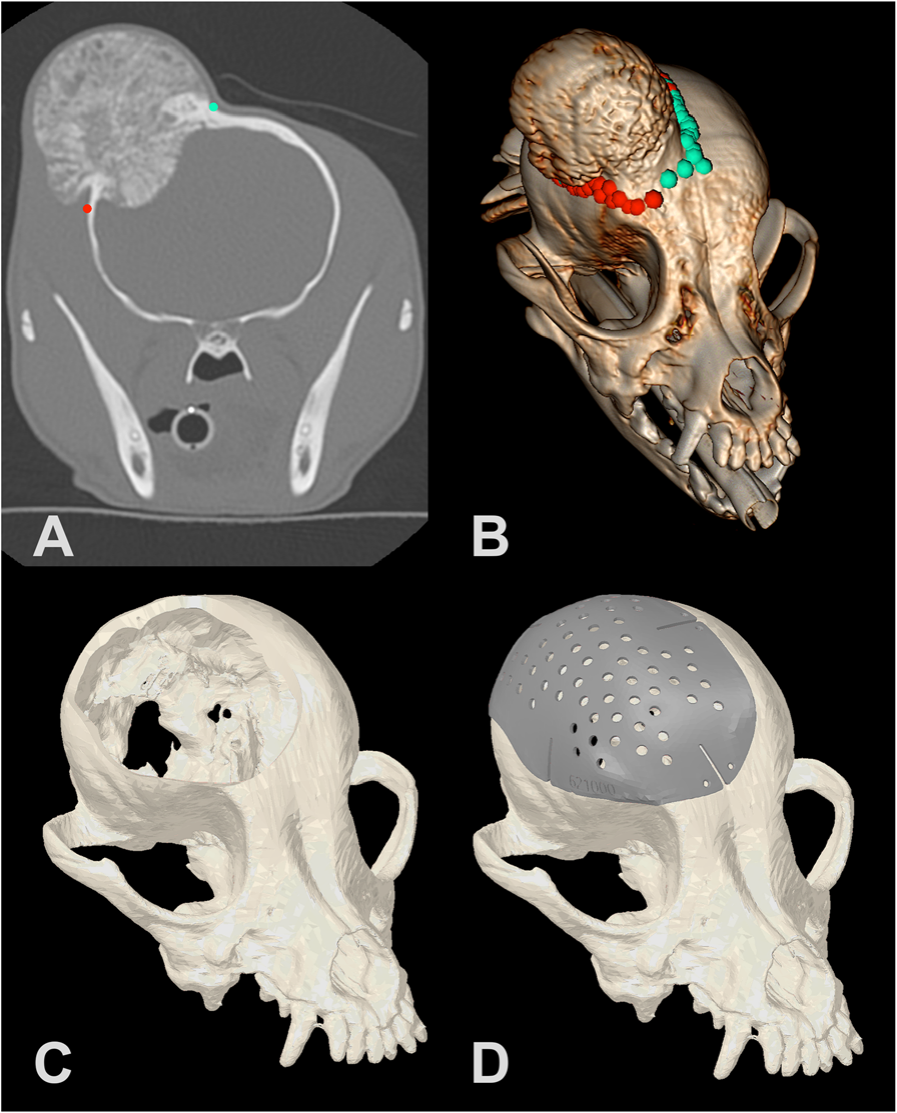
**a.** CT image with Region of Interest (ROI) points for Patient 2 in OsiriX MD imaging software. **b.** Three-dimensional reconstruction with ROI points in OsiriX MD. **c.** Three-dimensional spline in Geomagic Freeform software with tumor removed based on surgical cutting margins. **d.** Titanium plate designed in ADEPT software

Three-year-old, spayed female shih tzu/poodle cross that presented with a bony mass that was present for 2 years and 1 month prior to imaging. The CT scan revealed a 35 mm H × 25 mm W × 32 mm L, round, smoothly marginated, mildly lobulated, mineral dense, expansile mass arising from the calvarium, centered at the junction between the right parietal and frontal bones (Fig. 2a). The mass was non-homogenous with a coarsely granular and stippled appearance. The mass expanded into the right calvarium causing compression of the right parietal and frontal lobe, with no evidence of cerebral edema, and resulted in a mild left sided shift of the falx cerebri. The histological diagnosis was an osteoma.

### Patient 3

**Fig. 3:**
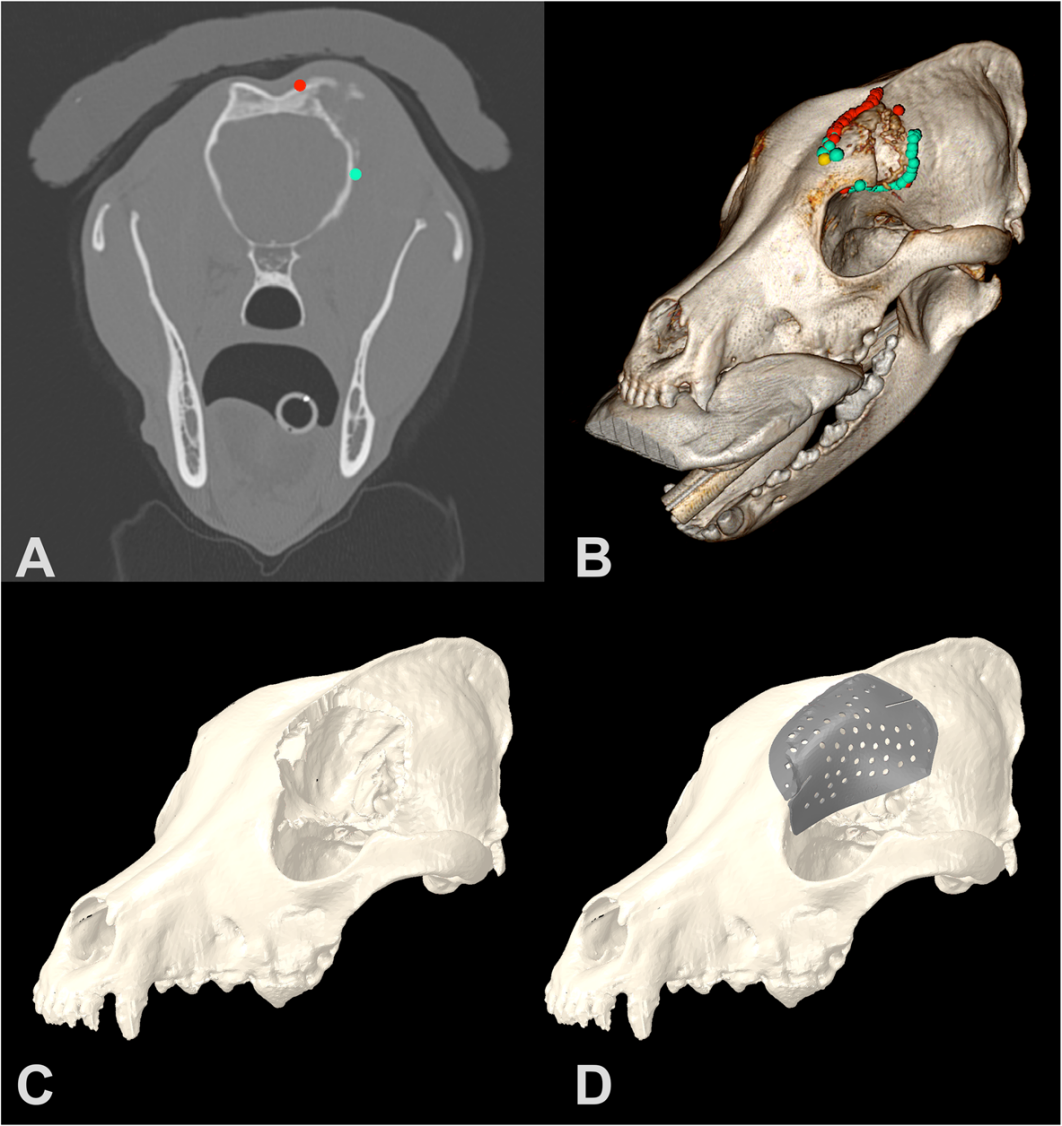
**a.** CT image with Region of Interest (ROI) points for Patient 1 in OsiriX MD imaging software. **b.** Three-dimensional reconstruction with ROI points in OsiriX MD. **c.** Three-dimensional spline in Geomagic Freeform software with tumor removed based on surgical cutting margins. **d.** Titanium plate designed in ADEPT software

Seven-year-old, spayed female Nova Scotia duck tolling retriever that presented with a firm mass on the skull that was first noted 14 days before imaging. The CT scan revealed a large mass arising from the left frontal bone overlying the left frontal sinus. The mass mildly contrast enhanced and invaded the left frontal bone, resulting in bone lysis and expansion of the bone, with mild smoothly marginated periosteal new bone formation, and a focal 2.5 mm defect between the lytic frontal bone and the brain (Fig. 3a). The mass extended into the dorsolateral aspect of the left frontal sinus. The diploe of the caudodorsal aspect of the frontal bone had an increased mineral density compared to the right side, that extended just to the right of midline. The mass (and mineral dense diploe) was 40 mm L, 28 mm W, × 29 mm H. The histologic diagnosis was a squamous cell carcinoma that invaded the calvarium and surrounding tissues.

All scans were imported into a DICOM viewer (Osirix MD, version 8.0.2, Pixmeo SARL, Bernex, Switzerland) and the tumors were confirmed to be primarily affecting and originating from the skull, based on CT imaging. The veterinary radiologist and surgical oncologist collaborated to identify the tumor margins in all cases using OsirixMD and the studies were exported for processing as .csv files as described (Fig. 1a-b, Fig. 2a-b, Fig. 3a-b). The tumor evaluation process from start to file export was performed with a median time of 642 s (240- 912 s), or just under 11 min. A 5 mm surgical margin was selected for demonstration purposes and a virtual defect created, as described, in all cases prior to plate creation (Fig. 1c, Fig. 2c, Fig. 3c). The median time to create the 3D spline and smooth the .csv contour was 5 min alone with an additional 5 min to create the tube for the margin cut from the spline. The plate was then designed in consultation with ADEISS using the software ADEPT, as described, with the engineer and surgical oncologist (Fig. 1d, Fig. 2d, Fig. 3d). The median time to create the cranial plate in ADEPT using the reconstructed skull with the craniectomy was 15 min. In all cases, margin identification, creation and plate design were completed within 2 h. ADEISS then printed and post-processed the cranial plate with a turnaround time of 2 weeks. In all cases, planning, printing, post-processing and shipping was completed within 3-weeks of CT scan image availability.

## Discussion

This study demonstrates that with access to advanced expertise and a medical metal 3D printing solution centre, such as ADEISS, patient-specific additive manufactured titanium implants for cranioplasty are feasible and can be manufactured for patient use within a 3-week turnaround from diagnosis to surgery. In 3 dogs, a patient-specific implant was created from CT images that were preoperatively planned, computer-designed and manufactured using the workflow outlined in this study.

Previous methods of cranioplasty in dogs are not ideal and recently the use of titanium mesh has been described in a small subset of dogs with good outcomes [1]. In humans it has been shown that titanium mesh can provide a safe and reliable implant that allows for postoperative CT and magnetic resonance imaging [13]. Despite this benefit, it does not always provide a cosmetic solution or one that reduces surgery time, speeds healing, or improves clinical outcome [6, 8, 9]. While none of these claims can be currently made for additive manufactured titanium plates, it can be hypothesized that some of these limitations may be eliminated with the use of this advanced technology. Access to patient-specific additive manufacturing for canine cranioplasty may allow for replacement of the previously preferred titanium mesh reconstruction in cases where complex contouring and reconstruction is necessary [1, 4, 14, 15]. In addition, these plates are designed to have a close fit to the remaining skull and can be fixed in place with traditional CMF screws, minimizing the need for additional specialized equipment.

This study relied on several different software programs and modalities for planning. Preoperative CT imaging is an important part of presurgical planning in patients with primary cranial tumors. The characteristics of the tumor on advanced imaging allow for determination of the invasiveness of the tumor and whether a patient is a surgical candidate or not [16]. The tumor margins were identified based on the CT scan in a DICOM viewer prior to transfer to CAD software for creation of the virtual defect. To identify the tumor margins, ROI points were marked on the CT with the radiologist and surgical oncologist and this method was the most easily translatable for communicating the exact location and extent of the tumor in the design phase. Creation and export of the tumor ROI allowed the design engineer to accurately create a defect with surgical margins that mimicked the surgical procedure and allowed for design of the implant. The surgical margin communicated to the design engineer directly related to the invasiveness of the tumor and extent of bone affected as seen in previous cases [17].

Access to a DICOM viewer should be readily available to most veterinary specialists who would be performing a surgery of this nature and it is important that the individuals involved in patient clinical care determine the tumor extent and planned surgical margins. Following identification of tumor extent and planned surgical margins, the rendering of the 3D skull, defect and plate design can be outsourced to an applications engineer or may be performed as part of a design and printing package with a 3D metal printing medical solution centre, when available. While we selected software that we are most comfortable using, the steps to create the 3D skull with the defect could be performed in many software programs that have ROI, segmentation and CAD capabilities.

For the design of the plate, we had access to a proprietary software, Renishaw ADEPT, which allowed for a seamless transition from the imported skull files to plate design. This study was able to demonstrate that despite the fact that this software was designed for use in human skulls, it was able to adapt to the many variations of the canine anatomy. By using this software, the team was able to design the plate in an efficient manner, allowing complete plate design and exportation for printing to occur in under 15 min. Alternatively, if the ADEPT software was not available, this whole process can be performed in a CAD software such as Geomagic Freeform which, in the authors experience, would require an advanced skillset and typically add approximately 2.5 h to the design process. Overall, while a general limitation of this technology is access and the skill and expertise needed to operate CAD software, where there is a limited knowledge base, most of the design process could be performed by application engineers. As metal printing becomes more readily available, it is likely that additional medical solutions centres will also provide support for this these types of procedures.

An important factor to consider when utilizing a pre-printed implant is the need for the resultant defect to fit the implant specifications. Accurate CT representation of the tumor was key in this study, as a pre-planned knowledge of the anticipated surgical defect was necessary to design the plate. Single-staged surgical planning and reconstruction is often necessary in veterinary medicine as a second surgical procedure may be cost prohibitive or unpalatable to owners. If a single-stage procedure is performed, careful planning is required to ensure that the previously designed implant will fit into the created defect. As a result, various methods need to be considered in order to standardize the surgical approach. When a single stage procedure is elected, intraoperative molding or patient-specific cutting guides have been used to aid in improving intraoperative accuracy [7, 18–22]. The use of a cutting guide is highly recommended in conjunction with the printed cranial plates discussed in this study. The use of patient-specific cutting guides in veterinary medicine has been sparsely reported but likely represent the future of surgical planning for advanced procedures and may aid in the accuracy of bone cuts and more accurate plate placement [10, 23–25].

The creation of a custom implant for patients in veterinary medicine with the use of additive manufacturing technologies has both benefits and pitfalls. Having access to a 3D metal printing medical solution centre, allows individuals to access this advanced expertise and service in a single location. A recent review of additive manufacturing in human medicine determined that the planning time costs for these types of surgeries is well worth the benefit, stating that an hour spent in production of an additive manufactured implant is equivalent in cost to 10 min saved in the surgical suite [26]. The use of patient-specific modelling has been proven in multiple studies to reduce surgical hemorrhage as well as reduce surgical time, which in effect decreases general anesthesia time and wound exposure [27]. The increased procedure time in human cranioplasty directly correlates with increased risk of surgical site infections which makes the importance of patient-specific implants that fit perfectly over the craniectomy site necessary [28]. Costs and cost savings for this procedure are unknown at this time but it is likely that this would result in a decrease in surgical and anesthesia times, and therefore costs may be similar to the current standard of care. Overall there are many potential benefits although the cost-benefit ratio has yet to be proven in veterinary medicine.

While the workflow and process described allows for access to this technology, there will be a lag of approximately 2 weeks from case submission to printed plate availability. In most cases, this planning time will not affect outcomes but may not be favorable to all owners. In addition, due to the highly specialized nature of these procedures, cost to the owner for the preoperative planning, surgery and postoperative recovery will still be a significant consideration. Despite these potential limitations, many patients that are already undergoing this complex procedure would ultimately choose to provide the best standard of care for the greatest outcome.

A limitation of the methods described in this paper is that CT imaging does not always accurately represent the full soft-tissue extent of the tumor making surgical margins more difficult to determine. The use of magnetic resonance imaging (MRI) may be helpful for evaluation of soft tissue components but was not considered in this study [25]. If MRI were included for surgical planning in the identification of tumor margins, a CT will still be necessary for this workflow process. In addition, depending on the availability of this modality, the inclusion of MRI may delay the planning process.

The plates created in this study were designed to cover the bony defect that would be created by surgical excision. In cases where intraoperative modification may be necessary based on the characteristics of the tumor, a staged procedure or creation of several plates may be necessary to allow more accurate planning of the implant after the defect is made. Due to the nature of these plates, it is possible to cut them to size in some regions, although care must be taken to ensure that there are still enough holes available for fixation. The creation of plates designed for more extensive intraoperative modification could be considered in future design iterations. A benefit of our design is that they utilize the same self-cutting, self-tapping screws as titanium mesh, therefore aside from the plate and guide, the surgical equipment necessary for this procedure is identical to traditional mesh cranioplasty procedures.

## Conclusions

This study demonstrated that a patient-specific additive manufactured implant may be created using this workflow and can be produced within a timeframe that is reasonable to ensure patient outcomes are not compromised. Having a patient-specific implant that fits securely on the skull and reconstructs the defect created following a craniectomy procedure may reduce surgery time, speed healing, and improve clinical outcomes for patients in the future. The ability to provide this type of care to patients undergoing a craniectomy has now been realized. Since this study was completed, the authors have successfully utilized the workflow and techniques in a clinical patient [29].

## Methods

This feasibility study was intended to investigate the workflow for the design and additive manufacturing of patient-specific titanium plates for canine cranioplasty. The process examined included computed tomography (CT) review through to implant received, no clinical patients received implants as part of this study.

### Image capture

CT scans from patients with primary skull tumors were evaluated. Cases were included if the tumor was predominately mineral dense and arose from the calvarium. CT images of the cranio-maxillary field were obtained using a 16-slice detector CT scanner (GE Brightspeed CT scanner, GE Healthcare, Milwaukee, Wisconsin, United States). The raw data were acquired with a standardized protocol in helical mode, 1.0-s rotation time, 0.562:1 pitch, 120 kV and 250 mA, 25-cm collimation, 512 × 512 matrix size, 0.488 mm in plane resolution, 0.625 mm through plane resolution using both standard and bone algorithms.

**Fig. 4:**
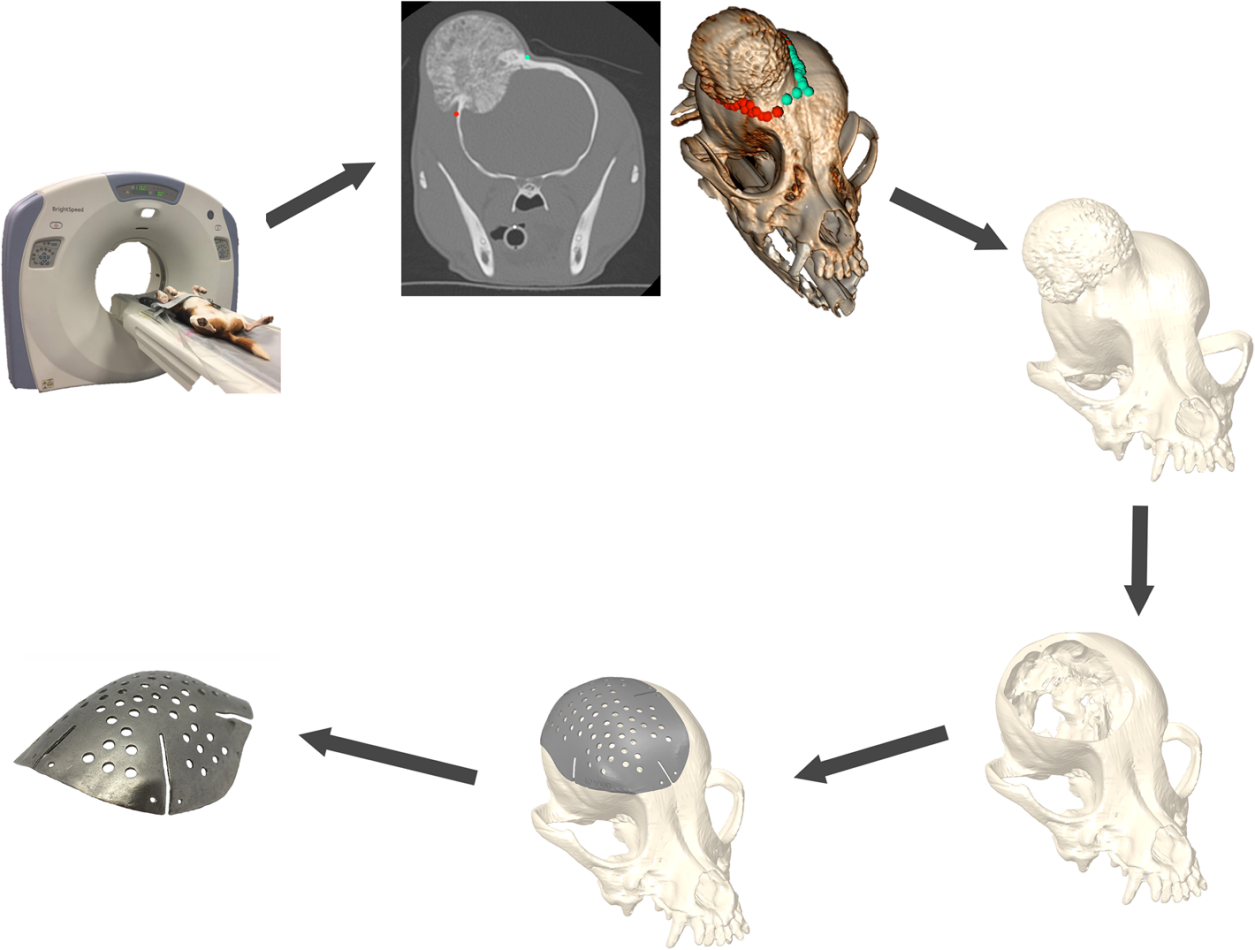
Proposed workflow from CT scan to printed plate. Figure created by J. James/M. Oblak/ RA ZurLinden AR zur Linden

Digital Imaging and Communications in Medicine (DICOM) format was used and the images were imported into a DICOM viewer (Osirix MD, version 8.0.2, Pixmeo SARL, Bernex, Switzerland). Tumor margins were determined based on agreement between a board-certified veterinary radiologist and board-certified veterinary surgical oncologist in Osirix MD. A bone algorithm and soft tissue algorithm were placed side by side for evaluation. A region of interest (ROI) point was placed at the most rostral extent of the tumor. ROI points were placed every 1 to 5 slices, with each slice being 0.625 mm. Each ROI point was placed on the left and right aspects of the tumoral margin (Fig. 4). The ROI points were placed superficially on the calvarium and point size was made based on evaluator preference. Once all points were placed, the image was converted to a 3D image using 3D volume rendering and a high contrast 3D preset to evaluate point placement and overall margin of the tumor (Fig. 4). An agreement was reached and the export ROI’s function was selected in order to export the data as a comma-separated values (.csv) file.

### Implant design and fabrication

The x,y,z point data from the .csv file were extracted and converted into a text file which was imported into ANSYS SpaceClaim (version 2017, ANSYS incorporated, Canonsburg, Pennsylvania, United States). A 3D spline was generated from the x,y,z point cloud data. The spline was smoothed to ensure the whole margin was still captured in the field of view. The DICOM files for the skulls were reconstructed using Materialise Mimics (version 19, Materialise NV, Leuvan, Belgium) and the stereolithography (.stl) file was exported using Materialise 3-matic.

The skull .stl and the 3D spline were imported into Geomagic Freeform (version 2017, 3D systems incorporated, Rock Hill, South Carolina, United States). The spline was given a radial thickness equal to the size of the cutting margin determined by the surgeon with the addition of the diameter of the surgical cutting burr. The thickened 3D spline was then subtracted from the skull to create the desired hole to be replicated for the craniectomy. The resulting skull model with defect was then exported as a .stl file and sent to Additive Design in Surgical Solutions (ADEISS) where they imported the file into the cranial plate creation software Additive-manufacture for Design-led Efficient Patient Treatment (ADEPT) (version 2017, Renishaw PLC, Wotton-under-Edge, Gloucestershire, United Kingdom). The cranial plate was designed in ADEPT software, with a video conference call between the veterinary surgical oncologist and application engineers to refine details including the margin overlap, perforations, plate thickness, slits, identifier embossing, screw hole sizing and placement over skull defect (Fig. 4). When the tumor did not cross midline, the plate contour was created by mirroring to the normal skull by the software. When necessary, the contour was refined by hand to visually match the normal skull shape.

At ADEISS, the plate file was imported into Computer Aided Manufacture (CAM) software QuantAM (version 2017, Renishaw PLC, Wotton-under-Edge, Gloucestershire, United Kingdom) in order to generate the additive manufactured support structure and manufacturing instruction file. This instruction file was sent to the AM400 (Renishaw) metal selective laser melting system and the plate was printed using medical grade titanium alloy (Ti-6Al-4 V) using a 40 um layer thickness. The completed plate was heat treated in an argon environment and then removed from the build substrate. The support structures were manually removed and the convex surface was finished to the roughness defined by the surgeon using a rotary tool. The plate was ultrasonically cleaned to remove all processing residues then shipped to the surgical facility for sterilization and surgery.

## Data Availability

The datasets used and/or analysed during the current study are available from the corresponding author on reasonable request.

## Abbreviations

CT: Computed tomography
DICOM: Digital imaging and communications in medicine
ROI: Region of interest
.stl: Stereolithography
